# New sorbent-based hydrophobic alginic acid derivatives for fat removal in multi-pesticide residues: analysis of a fatty food sample

**DOI:** 10.1039/d3ra07442k

**Published:** 2024-01-12

**Authors:** Omar A. Thabet, Fahad K. Alenzi, Maha A. Alshubramy, Khalid A. Alamry, Mahmoud A. Hussein, Richard Hoogenboom

**Affiliations:** a Department of Chemistry, Faculty of Science, King Abdulaziz University Jeddah 21589 Saudi Arabia kaalamri@kau.edu.sa mahussein74@yahoo.com maabdo@kau.edu.sa; b Saudi Food and Drug Authority Jeddah 22311 Saudi Arabia; c Chemistry Department, Faculty of Science, Assiut University Assiut 71516 Egypt; d Supramolecular Chemistry Group, Department of Organic and Macromolecular Chemistry, Centre of Macromolecular Chemistry (CMaC)Ghent University Krijgslaan 281 S4 9000 Ghent Belgium

## Abstract

Hydrophobic alginic acid derivatives were synthesized with various aliphatic hydrocarbon chains for fat removal in an analysis of multi-pesticide residues in a fatty food sample. First, alginic acid was chemically modified using eco-friendly ultrasound-assisted esterification with different alcohols, namely, hydrophobic alginic acid–methanol (HAA-C1), hydrophobic alginic acid–butanol (HAA-C4), and hydrophobic alginic acid–octadecanol (HAA-C18). The degree of esterification (DE) was determined by titration, and the results ranged from 57.3% to 63.7%. The physicochemical properties of the synthesized hydrophobic alginic acids (HAAs) were studied using FT-IR, XRD, TGA, and FE-SEM. Subsequently, the performance of the HAAs was checked and evaluated for the removal of fat from a fatty food sample by calculating the fat removal percentage and the determination of 214 pesticide residues in the fatty food sample. For the fat removal percentage application, the HAAs were able to efficiently remove between 77% and 83% of the fat; HAA-C18 had the highest percentage. Regarding the pesticide residue application, HAAs were also able to remove the fat content from the fatty food sample without a significant effect on the pesticide substances. The recoveries of the detected pesticide compounds were between 80% and 120% for all HAAs. However, there were various missing pesticide compounds for HAAs. The number of missing pesticide compounds was 19, 6, and 33 for HAA-C1, HAA-C4, and HAA-C18, respectively. HAA-C4 had medium hydrophobicity and it lost fewer pesticides than the other HAAs. This was because the multi-pesticide mixture had various classes of chemical structure; hence, it had different polarity powers. We concluded that HAAs are developable and applicable to be safely used as a green material in diverse fatty food sample analysis applications.

## Abstract 2

Hydrophobic alginic acid derivatives were synthesized with eco-friendly ultrasoundassisted esterification using various aliphatic hydrocarbon chains for fat removal in an analysis of multi-pesticide residues in a fatty food sample.

## Graphical abstract

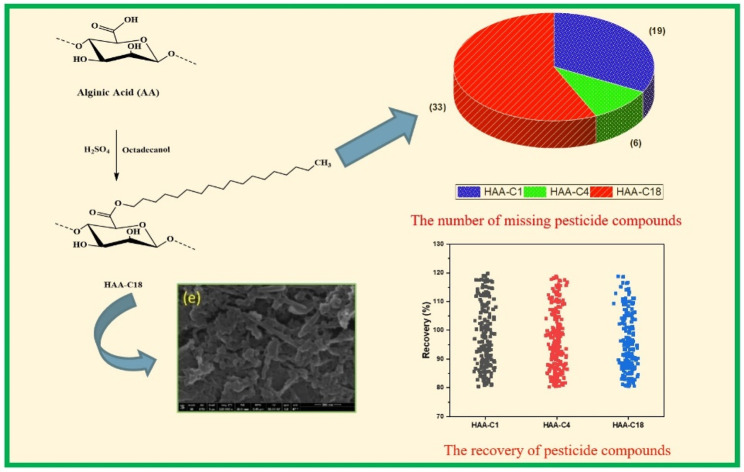

## 1. Introduction

Alginic acid is a heteropolysaccharide linear copolymer consisting of β-d-mannuronic acid (M) and α-l-guluronic acid (G) residues, which are connected through (1,4) glycosidic bonds and are arranged in either repeating GG (MM) blocks or alternating MG structures. Alginic acid is mainly derived from brown seaweed; it has been widely studied and used in different biomedical fields due to its remarkable characteristics, including its low cost, non-toxicity, superior biodegradability, and biocompatibility^1^,^2^. Despite the proven interest, alginic acid use remains limited due to the abundance of carboxyl and hydroxyl groups on the molecular chains. These dramatically increase the formation of intramolecular hydrogen bonds in alginate chains, making the structure rigid and stretched. The resulting difficult-to-load hydrophobic materials possess poor compatibility toward hydrophobic compounds. An abundance of hydrophilic groups decreases the stability of alginic acid in biological buffers, exhibiting unexpected and uncontrollable kinetic degradation and extensive water uptake properties^3,4^.

Recently, researchers have focused on the development of hydrophobic materials from alginates through covalent modifications; this field of research is rapidly growing. One approach involves the esterification of carboxyl groups by incorporating long-chain alkyl groups or aromatic rings. Alginates are then esterified using carbodiimide derivatives as a coupling reagent.^1^ Benzoyl chloride was used to substitute the hydroxy group with the benzoyl group.^2^ It is also possible to obtain alginate esters by treating tetrabutylammonium salts of alginic acid with alkyl halides. Alginates have been modified by direct esterification through Fischer esterification with selected alcohols in the presence of a suitable catalyst.^5^ However, the limited use of the Fischer esterification reaction is related to the degradation of the polysaccharide chain in the presence of strongly acidic conditions and high temperatures within the long-time reaction necessary to obtain a high degree of conversion. Research has been developed to overcome this limitation by decreasing the reaction temperature. Broderick *et al.* investigated the possibility of obtaining alginate butyl esters by applying sodium alginate, butanol, and H_2_SO_4_ as a catalyst at room temperature for 18 h.^6^ Murdzheva exploited ultrasonic irradiation to obtain methylated, ethylated, and isopropylated derivatives of alginic acid. Their finding revealed that an ultrasound-assisted synthesis performed at 45 kHz reduced the reaction time to 2 hours in contrast to esterification performed under conventional conditions of heating.^7^

There are many studies focused on the synthesis of lipophilic esters using polysaccharide biopolymers to enhance their properties and increase their potential for use in various applications.^8^ Chitin, cellulose, and agarose are other types of polysaccharide biopolymers that have been developed to obtain lipophilic esters. In chitin, chitin acyl ester compounds were synthesized with short and long chains of fatty acids to distinguish emulsifying,^9^ crystallinity, and thermal properties.^10^ Regarding the cellulose and cellulose derivatives, the modification by esterification process has taken place to produce lipophilic cellulose ester.^11^ In a previous study, carboxymethyl cellulose (CMC) was modified using various hydrocarbon chains by esterification *via* an eco-friendly ultrasound-assisted procedure to remove fat content in food analysis applications.^12^ In addition, cellulose ester was modified with extracted fatty acids from sunflower oil to be used as an additive in resins for vat photopolymerization.^13^ Agarose fatty acid esters are another modified biopolymer produced by the esterification process and are used as hydrocolloid surfactants to investigate their emulsifying ability.^14^ Also, agarose was modified using gallic acid to be used as coated films for seafood preservation in food packaging.^15^

Pesticides are multiple; various chemical groups have the ability to kill, repel, or mitigate animals, insects, or cultivated plant pests.^16^ Although pesticides save and protect crops and agricultural production worldwide, they pose a critical threat to human health when they are misused and can be transferred to the human body by consuming foodstuff.^17^ Therefore, there have been global collaborative efforts to limit the spread and misuse of pesticides; many regulations have been set for this purpose and monitoring methods have been increased/developed, whether *via* professional laboratories or farm inspections. Pesticides including insecticides, herbicides, and fungicides are classified into four main groups, based on their chemical composition, namely, organophosphate, organochlorine, pyrethroid, and carbamate.^18^ Their diversity in structure consequently affects the polarity strength of each group based on the attached function groups.

One of the most challenging issues in the detection of pesticides in foodstuff is the fat content in fatty food samples. Fat has several negative effects on the extraction process of pesticides as well as on the analytical instruments used, including contamination, carryover, and the blockage of columns or pipes.^19^ Many well-known techniques have been used to remove the fat from fatty food samples, for instance, liquid–liquid extraction with a strong non-polar solvent, Soxhlet extraction, microwave-assisted extraction, and freezing precipitation.^20^ Despite these attempts, there remains a requirement for a simple, low-cost, and efficient method to effectively perform the task. The QuEChERS (quick, easy, cheap, effective, rugged, and safe) method is a recent and widely used method used to extract pesticide residue from foodstuff,^21^ but it does not measure the fat content and is not able to clean the sample extractor of the fat content.^22^

Therefore, we aimed to synthesize a hydrophobic alginic acid to be used as a sorbent clean-up material to be then used in the pesticide residue detection of a fatty food sample. Initially, the chemical modification of alginic acid was conducted using eco-friendly ultrasound-assisted esterification with different alcoholic hydrocarbon chains. Subsequently, the efficiency of the fabricated material was evaluated by calculating the fat removal percent and by analyzing a real animal product sample, which was spiked with pesticide substances.

## 2. Experimental procedure

### 2.1 Materials and equipment

All solvents and chemicals used were of an analytical grade. Alginic acid powder (Sigma-Aldrich), methanol (Fisher; 99.8%), 1-butanol (Fisher; 99.8%), 1-octadecanol (Alfa Aesar; 97%), sulfuric acid (PanReac; 96%), ethanol (VWR; 99.7%), acetone (BDH; 99.5%), an LC multi-residue pesticide standard (RESTEK; P/N 31 971), a QuEChERS extraction kit (Agilent; P/N 5982-5650), DI water (Millipore; 18.2 MΩ cm), acetonitrile (ROMIL; 99.9%), formic acid (Sigma-Aldrich; 99%), and an ammonium format (VWR) were used in the experiment. Other equipment was used for the polymer synthesis and application procedure, including an ultrasonic water bath (Elmasnoic; S 60H), ESI-LC-MS/MS Triple Quad (Sciex 6500; Germany), an analytical balance (Mettler Toledo; 6 digit), a vortex (Fisher), a centrifuge (HERMLE; 13 000 rpm), and HPLC vials (Agilent; 2 mL). Regarding the characterization techniques, Fourier-transform infrared spectroscopy (FT-IR) (Thermo Scientific; Nicolet iS50 FT-IR) was used to identify the function groups. X-ray diffraction (XRD) (Bruker Co; D8 Discover; Cu target 40 kV; 40 mA; wavelength 1.54 A) was used to study the crystallographic structure. A thermogravimetric analysis (TGA) (Setaram; Themys one+) was used to study the response of the synthesized biopolymers to thermal stability. The samples were measured at a heating rate of 10° per minute under an argon atmosphere. Field-emission scanning electron microscopy (FE-SEM) (QUANTA FEG 205) was used to observe the microstructure image of the biopolymers.

### 2.2 Synthesis of hydrophobic alginic acid (HAA)

Alginic acid was modified using environmentally friendly ultrasound-assisted esterification with three different alcohols (methanol, butanol, and octadecanol). The synthesis of HAAs was adopted from previous work^7,23^. The reaction took place under heterogeneous conditions. Briefly, three conical flasks with caps were prepared, then 1 g alginic acid (AA) was weighed into each one. Next, 200 mL methanol was added to first sample (HAA-C1), 450 mL butanol was added to the second sample (HAA-C4), and 4.5 g octadecanol and 200 mL methanol as medium (1 : 1 mole) were added to the third sample (HAA-C18). The molar ratio of AA and all alcohols was 1 : 1. Subsequently, 1 mL sulfuric acid at a concentration of 96% was added to each sample, then all samples were placed in a constant ultrasonic water bath for 2 h at 40 °C. Finally, each new synthesized HAA was filtered then washed twice using 70% ethanol, followed by acetone. The new products were then dried in an oven at 40 °C for 1 h. The degree of esterification (DE) was determined by titration, as illustrated in the Food Chemical Codex.^24^ The physical and chemical properties of the synthesized biopolymers were checked using characterization tools (FT-IR, XRD, TGA, and SEM).

### 2.3 Determination of fat% removal

To investigate the performance of the synthesized hydrophobic AAs from a fat removal aspect, 2 g of chicken (a fat sample) was weighed in a 50 mL centrifuge tube, and then 10 mL of acetonitrile was added. After shaking for 2 minutes and centrifuging at 4000 rpm for 5 minutes at room temperature, the liquid layer (acetonitrile) was transferred to another tube and evaporated under the N_2_ evaporation system until dryness. The remaining fat matrix was weighed and recorded, then dissolved again using 10 mL of acetonitrile. At this stage, the HAAs were added, shaken for 10 minutes, and centrifuged again in the same condition as mentioned above to precipitate the HAAs with fat matrix content. After that, the acetonitrile layer was moved to a new tube and then evaporated until dryness, and the remaining fat was weighed and recorded again. The fat% removal was calculated by dividing the wt fat after using HAAs over the wt. fat before, then multiplying by 100. This process was repeated three times, and the average percentage was reported for each HAA.

### 2.4 Determination of pesticide residues in a fat sample using HAAs

The sample preparation and the extraction of pesticide residues from a fat sample were adopted from an international method to minimize the error sources.^25^ Briefly, 2 g of a homogenized chicken sample was weighed in a 50 mL centrifuge tube and spiked with 30 μg kg^−1^ of a mixed pesticide standard. Subsequently, 10 mL acetonitrile was added and shaken for 2 min. The extraction salt (QuEChERS) was then added to the mixture and it was manually shaken for 2 min, then centrifuged at 4000 rpm for 5 min at room temperature. Next, 1 mL of the extractor (upper layer) was transferred to a new 2 mL Eppendorf tube. HAA biopolymers were added to remove the fat content. The mixture was shaken for 5 min, then centrifuged again for 5 min at 4000 rpm at 4 °C. Finally, the upper layer was moved to an HPLC vial and injected into LC-MS/MS.

The analysis was performed using a liquid chromatography-mass spectrometer 6500 triple quad instrument (±ESI-LC-MS/MS) and a 1290 Infinity UPLC system with ideal conditions. The analytical column used was reverse phase (C18; 2.1 × 150 mm; 1.8 μm). The mobile phase consisted of water; each had a 5 mM ammonium format with 1% formic acid. The MS parameters were set for optimized conditions, with an ion source temperature of 550 °C, ion spray potential of 5500 V, and input potential of 10 V. The quantification of the measured pesticides was calculated using a solvent calibration curve (external calibration strategy).

## 3. Results and discussion

### 3.1 Chemistry

**Scheme 1:**
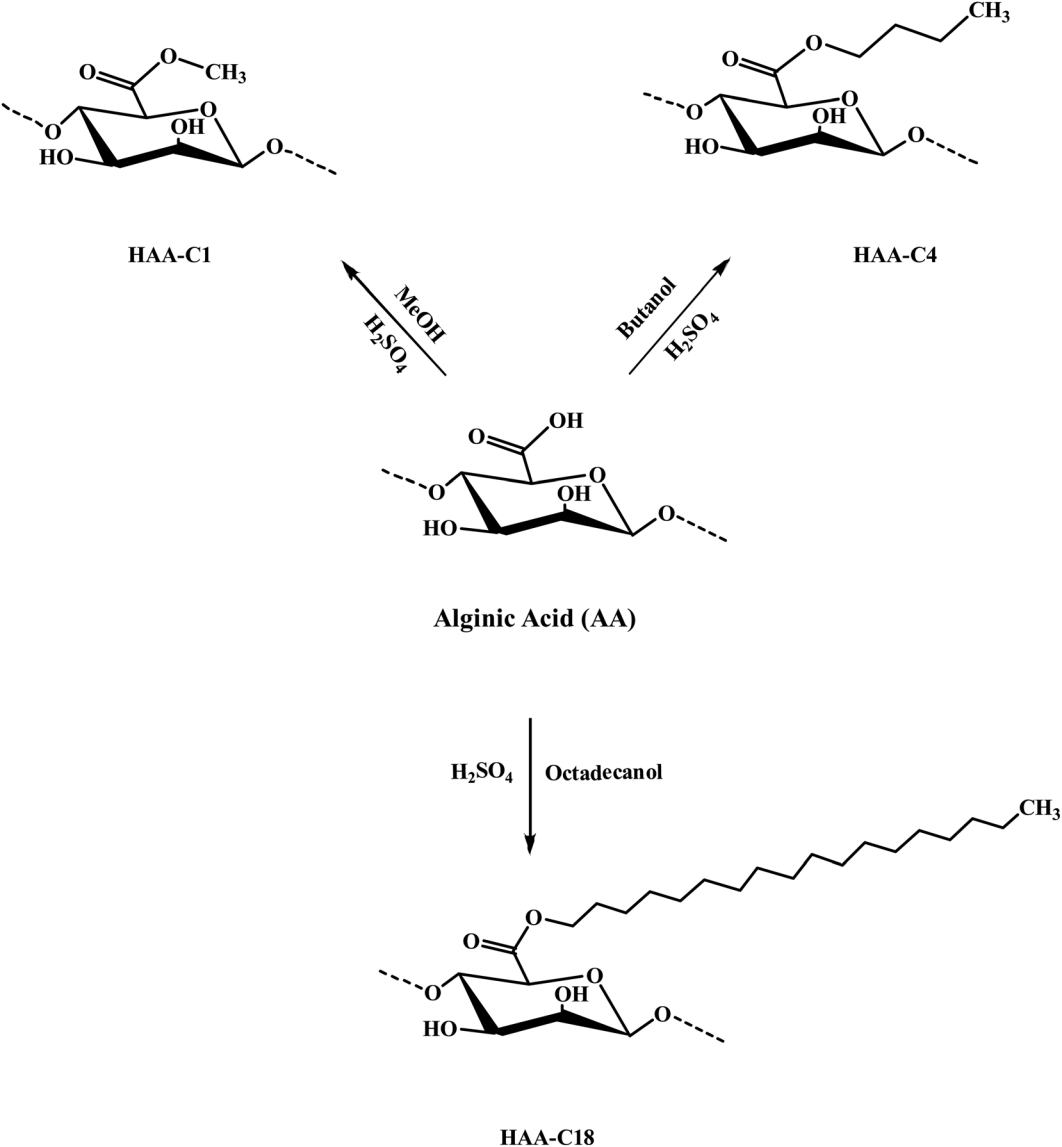
Preparation mechanism of the modified hydrophobic alginic acids HAA-C1, HAA-C4, and HAA-C18.

The modified ester of alginic acid HAAs was successfully synthesized using a simple, fast, cost-effective, and eco-friendly method by combining acid catalyzed with ultrasound-assisted irradiation. Methyl, butyl, and octadecyl alginates were esterified, as shown in Scheme 1. The heterogeneous synthesis of HAAs in the (methanol/H^+^) depended on the surface interaction between the solid phase (alginic acid) and the liquid medium (methanol/H^+^). The use of ultrasound in the irradiation process significantly reduced the reaction time from 72 h to just 2 h.^7^ The ultrasound also generated gas microbubbles that implosively disrupted, forming cavitation bubbles at incredibly high temperatures and pressures. These actions resulted in a significant acceleration of the reaction. In HAA-C18, methanol was used as a medium because the octadecanol is in powder form as well as alginic acid, so there is a possibility of esterification with methanol instead of octadecanol. In spite of that, octadecanol competed with methanol in this interaction due to its higher molecular weight. FT-IR spectroscopy supported this interpretation. Thereafter, the degree of esterification of AA was determined and provided at 63.7 ± 4.6%, 57.3 ± 2.7%, and 59.4 ± 3.1% for HAA-C1, HAA-C4, and HAA-C18, respectively.

### 3.2 Characterization confirmation

#### 3.2.1 FT-IR analysis

**Fig. 1:**
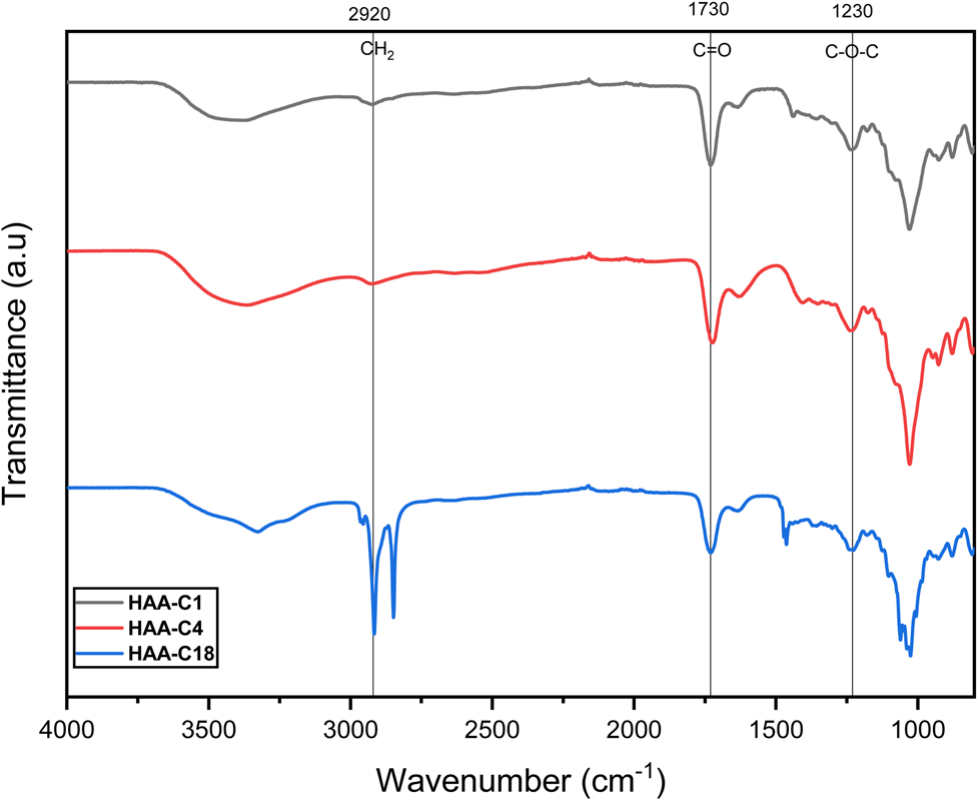
FT-IR spectra of the modified hydrophobic alginic acids HAA-C1, HAA-C4, and HAA-C18.

The successful synthesis of HAAs was verified by FT-IR spectra, as shown in Fig. 1. The FT-IR spectra of HAA-C1, HAA-C4, and HAA-C18 were compared with the AA spectrum reported in a previous work.^26^ HAA-C1, HAA-C4, and HAA-C18 exhibited additional weak bands apart from the basic characteristic absorption bands of AA. The absorption bands of HAA-C1 and HAA-C4 at 2930, 2927, and 2926 cm^−1^ significantly enhanced HAA-C18, representing the stretching vibration of –CH_2_ on the methyl, butyl, and octadecyl groups. Extra bands appeared at 1734, 1733, and 1740 cm^−1^, owing to the stretching vibration of C=O on the ester groups. The other new signals that appeared at 1245, 1243, and 1244 cm^−1^ were attributed to the stretching vibration of C–O on the ester group. These results indicated that the methyl, butyl, and octadecyl groups had successfully grafted onto alginate *via* the esterification reaction to generate the HAAs.

#### 3.2.2 X-ray diffraction (XRD) analysis

**Fig. 2:**
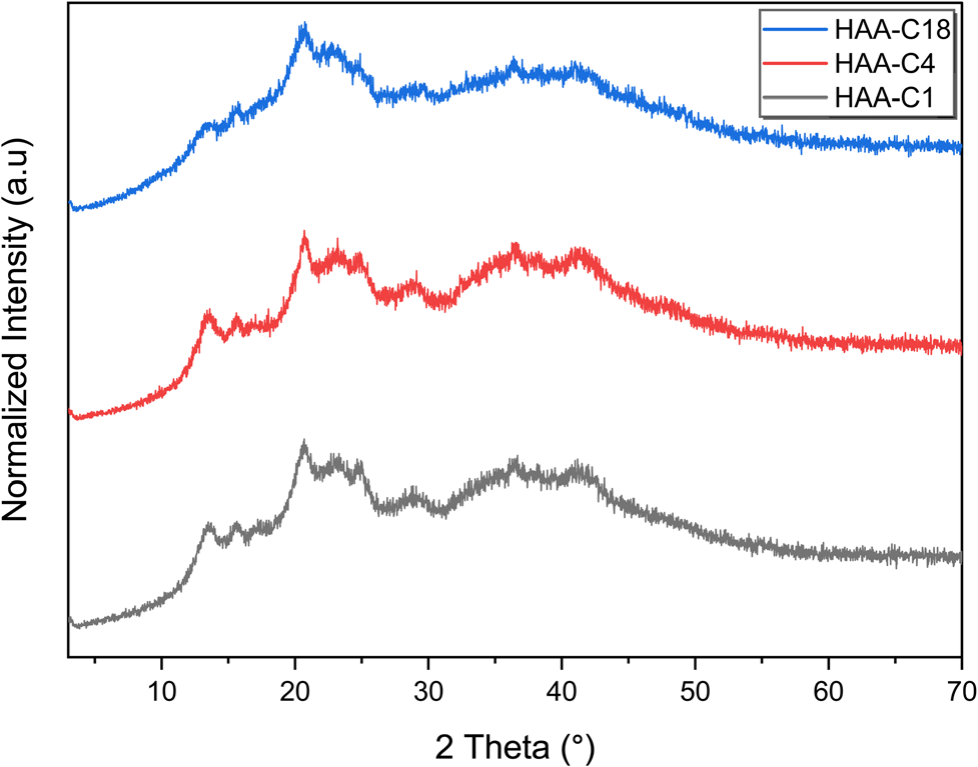
XRD curves of the modified hydrophobic alginic acids HAA-C1, HAA-C4, and HAA-C18.

XRD is the most direct and effective method to analyze the change of crystal structures of alginates in esterification reactions. As shown in Fig. 2, HAA-C1, HAA-C4, and HAA-C18 exhibited weak crystalline diffraction peaks, indicating their amorphous structures. Diffraction peaks of AA at 13.4° and 22.7° are typical characteristic peaks of hydrated crystalline structures, resulting from the intermolecular hydrogen bonds of AA.^27^ HAA-C1, HAA-C4, and HAA-C18 revealed a sharp diffraction peak at 2*θ* = 14.8° and a wider diffraction peak at 2*θ* = 20° after the chemical modification, similar to the previously reported structure characteristics of alginate derivatives.^3^ Hence, the transformation of these crystal diffraction peaks for AA indirectly indicated that the esterification modification had weakened and destroyed the intramolecular hydrogen bonds of the alginate, thus enhancing its molecular flexibility.^28–30^

#### 3.2.3 Thermal gravimetric analysis (TGA)

**Fig. 3:**
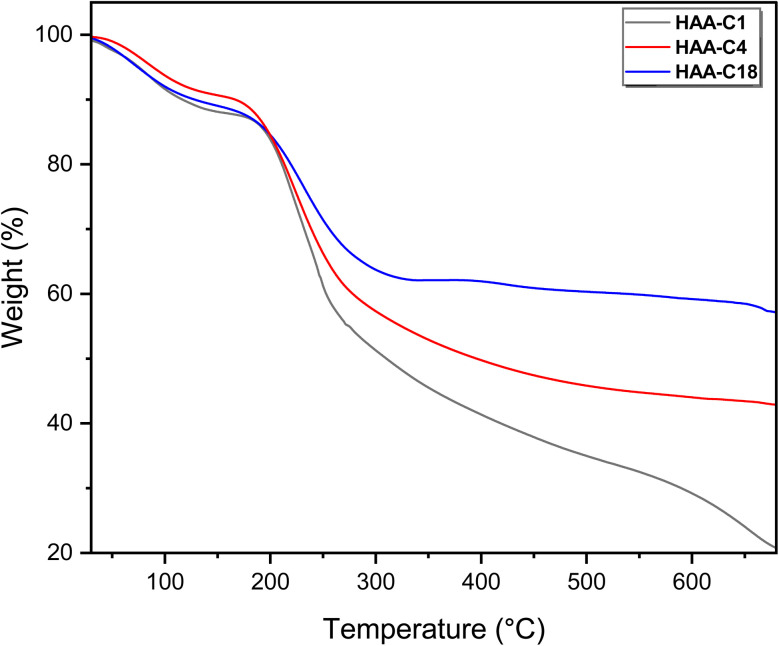
TGA thermograms of the modified hydrophobic alginic acids HAA-C1, HAA-C4, and HAA-C18.

A thermogravimetric analysis was used to estimate the different thermal stabilities of alginic acid as well as the aliphatic ester derivatives. The measurement has been carried out at a heating rate of 10° per minute under an argon atmosphere. The mass loss curves of HAA-C1, HAA-C4, and HAA-C18 are presented in Fig. 3. From the thermograms, we observed that all three materials exhibited different thermal behaviors, indirectly reflecting the change in molecular structures. The TGA of the alginic acid AA curve was reported as three consecutive weight-loss steps. The first stage of weight loss, at approximately 16.05% of the initial weight, was probably due to intermolecular water molecules from the polymer.^31^ The IDT abbreviation represents the initial degradation temperature at which the decomposition starts. All the measured materials showed nearly similar IDT values (195–200 °C), which are higher values compared to the pure AA sample. Fig. 3 demonstrates that the weight losses of the first stage of HAA-C1, HAA-C4, and HAA-C18 were approximately 13%, 9%, and 11%, respectively. Similar degradation behavior was observed for the IDT results, as illustrated in Table 1. This phenomenon was a direct effect of the hydrophobic nature of the introduced aliphatic groups, which caused a reduction of intermolecular water in the alginic acid.^2^

**Table 1:** IDT, FDT, and temperature at 10%, 20%, 30%, and 40% of weight loss, extracted from TGA analysis^a^

Biopolymer	IDT	FDT	Temperatures derives weight loss%			
			10%	20%	30%	40%
AA^31^	170	Completely decomposed	100	195	280	300
HAA-C1	200	∼700	110	200	225	250
HAA-C4	195	>700	160	200	230	280
HAA-C18	200	>700	120	220	255	>700

a IDT: initial decomposition temperature; FDT: final decomposition temperature.

The second weight loss occurred at an inflection point of 200 °C. This degradation step was the main step (fastest step) for all modified derivatives (HAA-C1, HAA-C4, and HAA-C18). This behavior designated the decomposition of the alginate by dehydrating the saccharide rings, breaking the C–H and C–O–C glycoside bonds in the main polysaccharide chain. The data in Table 1 demonstrate the thermal behavior of the modified structure, where 10%, 20%, 30%, and 40% represent the weight loss. No significant change between AA and HAA-C1 was observed at 10%; at 20% to 40%, the degradation decreased with an increase in temperature as the side chain of ester rose. HAA-C4 and HAA-C18 modified with a flexible side chain showed steady state thermal stability started at 450 and 300 °C, respectively, compared with AA and HAA-C1, which had fewer thermal stability characteristics.

Finally, the thermograms showed a highly thermal stable structure in the third degradation step of HAA-C18, caused by the decrease in the carboxyl groups of the polymer and the formation of ester bonds that destroyed the polymeric intramolecular hydrogen bond. All the outcomes confirmed that the esterification of alginic acid was successfully introduced. The final degradation temperature (FDT) refers to the temperature at which the decomposition is completed or becomes final. The literature confirms that pure AA is completely decomposed at 700 °C.^31^ Whereas, the other materials show significantly higher FDT values in comparison to pure AA. The FDT values are in the following order: HAA-C1 = HAA-C4 < HAA-C18. Such observations are attributed to the measurement conditions under an argon atmosphere, which reduce the thermal degradation process.

#### 3.2.4 Morphological analysis

**Fig. 4:**
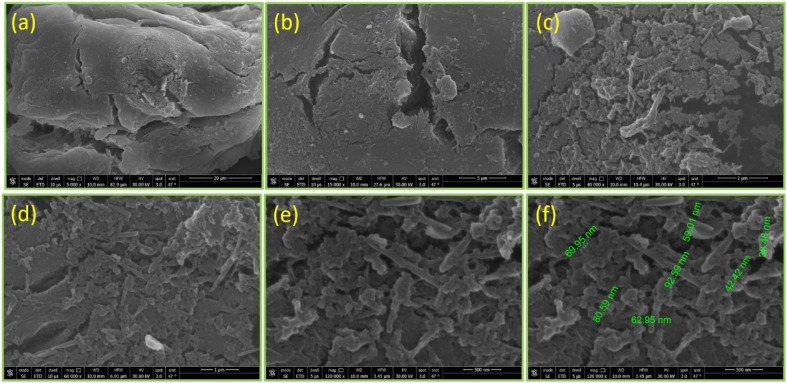
SEM images of HAA-C18 morphology at different magnifications: (a) at 5000×; (b) at 15 000×; (c) at 40 000×; (d) at 60 000×; and (e and f) at 120 000×.

The TGA study of HAA-C18 was found to have high thermal stability over a range of temperatures. To investigate this stability, the morphologies of HAA-C18 were studied using scanning electron microscopy (SEM) at various magnifications. From the micrographs (shown in Fig. 4), the surface was less folded and the folding pattern roughness displayed a porous surface with a network of crevices and protrusions, observed more at higher magnifications. At lower magnifications (Fig. 4(a and b)), the surface morphology appeared to comprise irregular semi-spheroids with uneven surfaces and microscopic pores. At higher magnifications (Fig. 4(c and d)), the surface exhibited short and irregular fibers, with an approximate width of 0.25 μm. At extremely high magnifications of approximately 120 K (Fig. 4(e and f)), the surface showed an aggregation of short, flat, and non-uniform strips, approximately 92.59 to 38.48 nm. Regular patterns have been reported from alginic acid as a folded surface structure;^2^ hence, all the changes seen in the obtained morphology confirmed the esterification of alginic acid. The modifications resulted from a shift in the internal order, which influenced the polymer crystal structure with heterogeneous molecules between the polymeric alginic acid chains and the immobilization of the aliphatic chain with glucose hydroxyl groups on the repeating polysaccharide units.

### 3.3 Fat% removal application

The physical appearance of the final extractor was an initial indicator of HAAs working; it became clearer and finer after applying HAAs in the extraction procedure. HAAs were able to remove approximately 77–83% of the fat in the clean-up step. HAA-C18 had the highest removal percent of approximately 83%; the lowest was achieved by HAA-C4. Table 2 shows the fat% removal of each HAA. In general, all HAAs were able to efficiently remove fat and the results were close to each other. Therefore, the results were accepted for the intended purpose, and are promising for future developments.

**Table 2:** The fat% removal of HAAs biopolymers

Hydrophobic alginic acids	Wt fat (mg)		Removal (%)
	Before	After	
HAA-C1	5.00	0.93	79.4
HAA-C4	4.51	1.01	77.6
HAA-C18	4.51	0.89	82.2

### 3.4 Pesticide residue application

The ability of the fabricated HAAs to remove fat from the sample was checked using HAAs as a clean-up in a pesticide residue application of a fatty sample. Pesticide compounds were chosen due to their diversity of chemical structures; they have wide groups of semi-polar and non-polar substances. Therefore, this method was appropriate to investigate the performance of HAAs to rend off the fat without affecting the extraction and recovery of analytes.

**Fig. 5:**
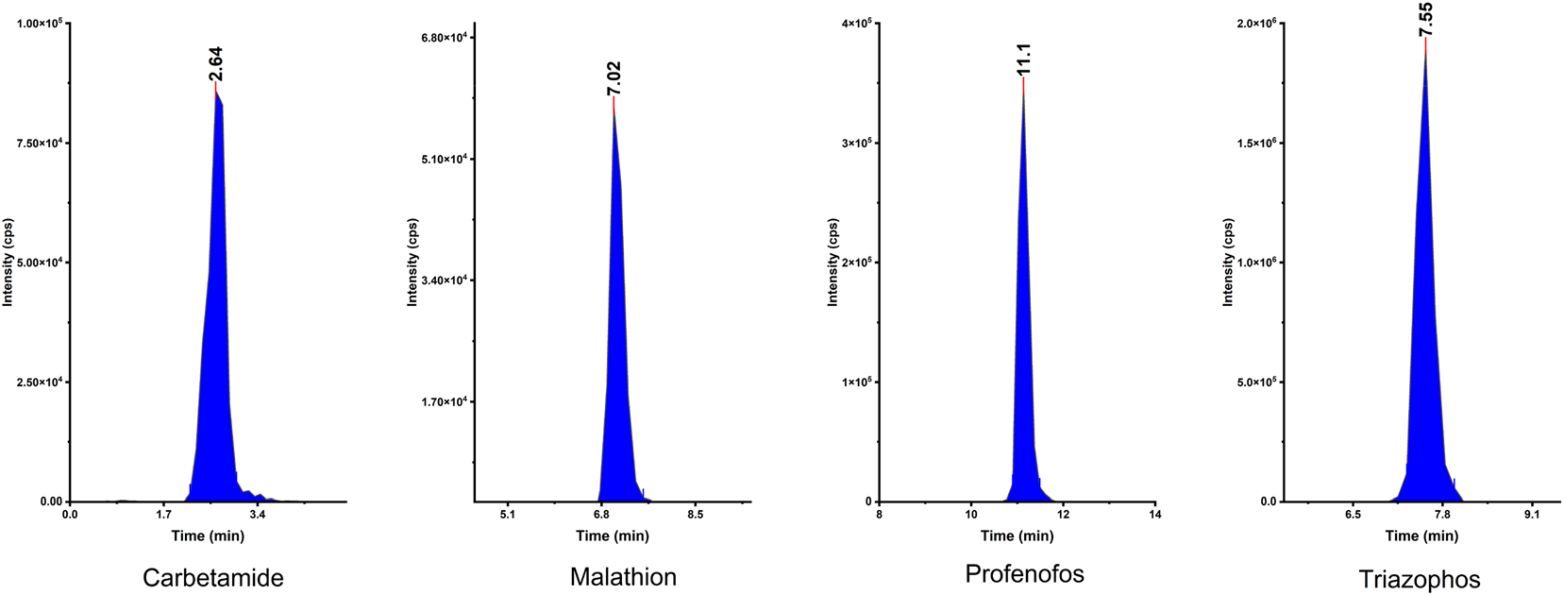
The chromatograms of peak shapes for a few pesticide compounds.

An LC mixture standard containing 214 compounds from various chemical pesticide classes such as organochlorine, organophosphorous, carbamate, and pyrethroid was used and spiked in the fatty animal product sample. First, pesticide compounds were analyzed without the HAAs to check their stability and activity. Subsequently, HAAs were applied as a clean-up step and the results were evaluated. Fig. 5 shows the integrated peak shapes for a few of the representative pesticide compounds.

**Fig. 6:**
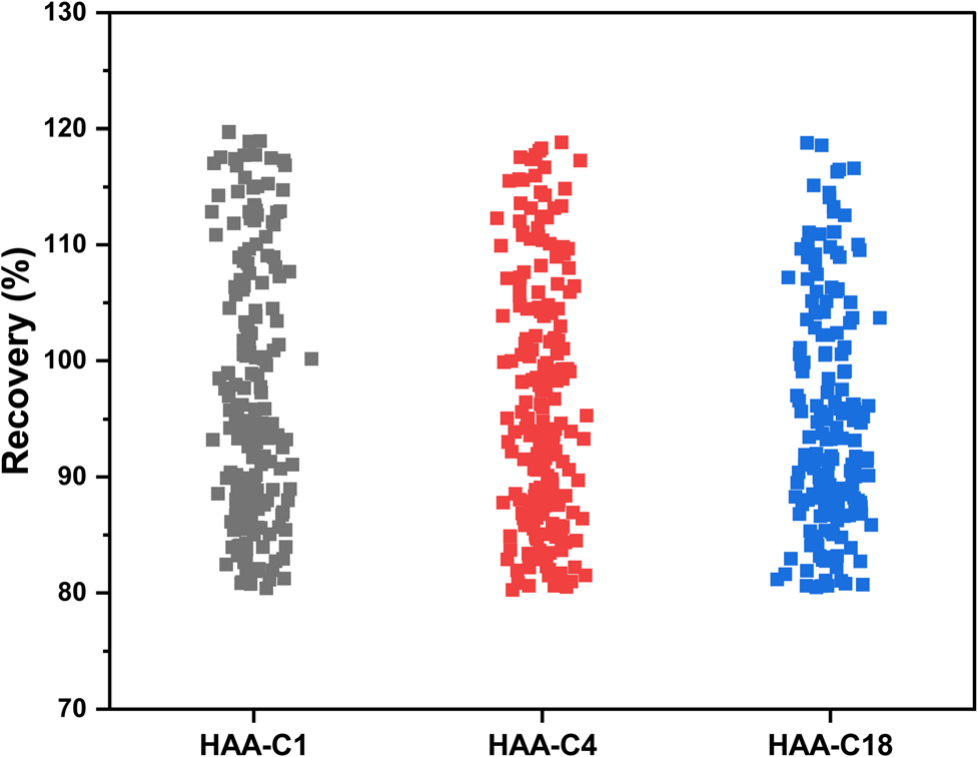
The recoveries of pesticide compounds after using HAAs.

**Fig. 7:**
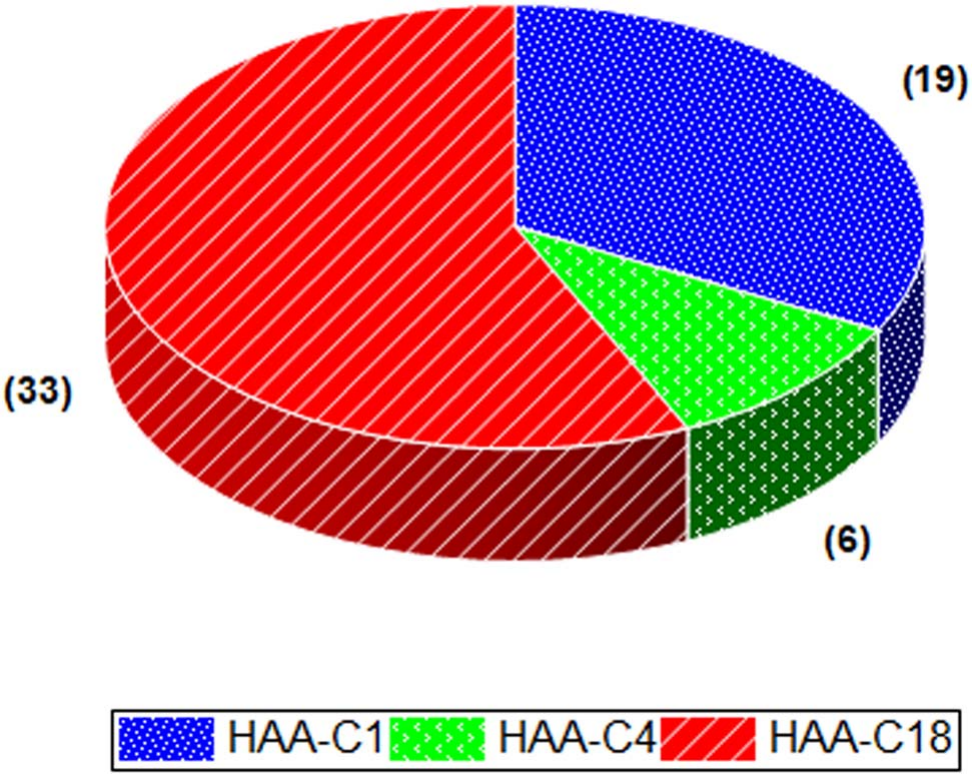
The number of missing pesticide compounds after using HAAs.

The effect of HAA-C1, HAA-C4, and HAA-C18 on the pesticide compounds was individually evaluated; the analyte recoveries are reported in Table 3. All HAAs provided satisfactory recoveries for the detected pesticide compounds. The recovery ranged from 80% to 120%, which met the requirement specifications according to SANTE/2017.^23^Fig. 6 illustrates the recovery ranges for the pesticide compounds after applying the three HAAs. There were various missing pesticides after using HAAs as a fat sorbent. The number of missing pesticide compounds were 19, 6, and 33 for HAA-C1, HAA-C4, and HAA-C18, respectively, as displayed in the pie chart in Fig. 7. The un-unified missing pesticide compounds among HAA sorbents could be interpreted as being due to the difference in the chemical interactions between pesticides and HAAs. The highest number of missing compounds appeared after using HAA-C18, where 33 pesticides were lost (including 4,4′-dichlorobenzophenon, cycloxydim, and carbaryl). After checking the structure of the missed pesticide compounds, it was clearly observed that they had high hydrophobicity properties that could chemically interact with the most hydrophobic HAA-C18; hence, they were probably removed with the fat in the clean-up stage. HAA-C1 and HAA-C4 which had short hydrocarbon chains and, hence, fewer hydrophobicity properties—lost fewer pesticide compounds. It was observed that HAA-C1 lost a greater number of compounds than HAA-C4 (*e.g.*, flufenoxuron), possibly due to the high polarity. In conclusion, HAAs could be used as an efficient removal sorbent for fat in pesticide residue applications, but we highly recommend that the various structures of the wide groups of pesticide compounds and the consequential effects are considered.

**Table 3:** The result of recovery study for pesticide compounds after using HAAs

Pesticides name	Spiking level (μg kg^−1^)	Average recovery (%) (±CV% for *n* = 3)		
		HAA-C1	HAA-C4	HAA-C18
(Monceren) pencycuron	30	ND	82.3 ± 4	80.8 ± 4
3,4-Dichloroaniline	30	106.5 ± 7	106.3 ± 7	103.3 ± 7
4,4′-Dichlorobenzophenon (4.4-DBP)	30	ND	83.4 ± 6	ND
Acibenzolar-*S*-methyl	30	82.9 ± 6	82.2 ± 6	ND
Aclonifen	30	88.6 ± 12	86.8 ± 12	88.3 ± 12
Ametryn	30	82.1 ± 9	85.1 ± 10	ND
Amitraz	30	92.6 ± 9	93.0 ± 10	85.9 ± 9
Atrazine	30	82.5 ± 9	82.0 ± 9	ND
Azaconazole	30	104.3 ± 18	104.8 ± 17	99.0 ± 18
Azinphos-methyl	30	85.1 ± 13	84.6 ± 13	83.2 ± 10
Azoxystrobin	30	99.0 ± 11	97.5 ± 12	95.0 ± 12
Beflubutamid	30	112.9 ± 9	102.2 ± 6	93.2 ± 4
Benalaxyl	30	100.2 ± 2	101.8 ± 6	86.7 ± 5
Bendiocarb	30	91.3 ± 12	88.4 ± 11	99.7 ± 13
Bentazone	30	89.6 ± 17	98.3 ± 19	91.1 ± 18
Benzovindiflupyr	30	113.3 ± 8	107.7 ± 8	95.5 ± 8
Benzoximate	30	98.8 ± 18	89.4 ± 16	96.6 ± 18
Boscalid	30	115.1 ± 15	115.6 ± 15	112.9 ± 15
Bromacil	30	93.4 ± 14	92.6 ± 18	89.3 ± 17
Bromfenvinfos	30	82.7 ± 13	85.8 ± 13	91.5 ± 14
Bromucanozole isomer	30	103.1 ± 13	96.3 ± 12	96.3 ± 12
Bupirimate	30	88.9 ± 21	81.0 ± 20	80.6 ± 19
Butafenacil	30	117.5 ± 13	114.3 ± 13	104.2 ± 12
Butylate	30	85.5 ± 12	83.7 ± 12	ND
Carbaryl	30	81.3 ± 14	81.5 ± 14	ND
Carbetamide	30	95.8 ± 22	95.1 ± 22	90.4 ± 21
Carbofuran	30	89.9 ± 18	88.6 ± 18	89.3 ± 18
Carboxin	30	84.1 ± 20	86.5 ± 20	80.7 ± 19
Carfentrazone-ethyl	30	107.3 ± 18	111.5 ± 19	105.1 ± 18
Chlorantraniliprole	30	100.4 ± 23	99.9 ± 23	95.9 ± 22
Chlorbromuron	30	94.0 ± 21	92.0 ± 20	90.8 ± 20
Chlorfenvinphos	30	81.9 ± 10	87.7 ± 11	88.5 ± 11
Chlorfluazuron	30	104.5 ± 12	97.9 ± 11	90.5 ± 10
Chlorotoluron	30	90.4 ± 14	91.6 ± 14	86.8 ± 14
Chloroxuron	30	93.9 ± 17	95.6 ± 17	91.8 ± 16
Chlorpropham	30	89.9 ± 3	88.1 ± 3	88.2 ± 3
Chlorpyrifos-methyl	30	89.0 ± 15	90.6 ± 15	81.2 ± 13
Chlortoluron	30	91.7 ± 14	92.0 ± 14	86.8 ± 14
Clethodim isomer	30	ND	80.6 ± 11	ND
Clodinafop-propargyl	30	117.8 ± 13	110.5 ± 12	104.1 ± 12
Clofentezine	30	87.6 ± 2	86.5 ± 2	ND
Clomazone	30	94.9 ± 12	93.7 ± 12	90.6 ± 12
Cloquintocet-mexyl	30	89.0 ± 11	88.1 ± 11	ND
Coumaphos	30	86.8 ± 17	94.8 ± 18	96.0 ± 18
Cyantraniliprole	30	113.0 ± 11	109.8 ± 11	110.0 ± 11
Cyazofamid	30	84.0 ± 9	91.6 ± 9	81.6 ± 8
Cycloate	30	ND	ND	80.6 ± 18
Cycloxydim	30	86.1 ± 13	80.6 ± 12	ND
Cycluron	30	95.9 ± 19	93.1 ± 18	90.0 ± 18
Cyflumetofen	30	110.7 ± 3	107.2 ± 3	114.5 ± 3
Cymoxanil	30	97.7 ± 10	83.7 ± 9	87.0 ± 9
Cyproconazole isomer	30	100.2 ± 18	93.9 ± 22	89.0 ± 21
Cyprodinil	30	82.9 ± 19	90.6 ± 21	82.8 ± 19
Desmedipham	30	97.7 ± 19	98.4 ± 20	91.2 ± 18
Diazinon	30	93.2 ± 13	86.9 ± 12	ND
Dichlorvos	30	93.2 ± 9	94.2 ± 12	89.1 ± 10
Diclobutrazol	30	81.2 ± 20	83.4 ± 20	87.4 ± 21
Diethofencarb	30	109.7 ± 8	110.9 ± 5	105.2 ± 7
Difenoconazole isomer	30	100.2 ± 17	102.0 ± 17	95.6 ± 16
Difenzoquat metilsulfate	30	91.1 ± 13	91.3 ± 13	89.7 ± 13
Diflubenzuron	30	114.3 ± 22	112.4 ± 22	102.4 ± 20
Dimethachlor	30	94.6 ± 13	95.3 ± 13	91.1 ± 13
Dimethomorph isomer	30	ND	117.4 ± 27	116.5 ± 27
Dimoxystrobin	30	96.1 ± 12	91.0 ± 15	88.6 ± 11
Diniconazole	30	85.7 ± 18	93.4 ± 20	88.2 ± 19
Disulfoton sulfoxid	30	93.4 ± 13	94.0 ± 13	91.4 ± 13
Edifenphos	30	80.9 ± 12	80.5 ± 12	ND
EPN	30	112.8 ± 19	103.9 ± 15	90.1 ± 20
Epoxiconazole	30	107.0 ± 13	99.3 ± 14	96.5 ± 14
Etaconazole isomer	30	93.6 ± 4	104.6 ± 8	95.9 ± 5
Ethion	30	85.0 ± 4	81.7 ± 4	80.5 ± 3
Ethofumesate	30	108.9 ± 7	103.9 ± 6	106.3 ± 6
Ethoprophos	30	88.0 ± 14	87.5 ± 14	87.0 ± 13
Fenamidone	30	100.3 ± 20	100.6 ± 20	ND
Fenamiphos	30	106.1 ± 11	101.5 ± 14	101.1 ± 12
Fenamiphos-sulfoxide	30	101.4 ± 9	101.0 ± 9	95.3 ± 8
Fenarimol	30	112.0 ± 14	109.7 ± 14	111.1 ± 14
Fenbuconazole	30	ND	114.6 ± 13	103.7 ± 11
Fenhexamid	30	106.7 ± 23	ND	112.6 ± 24
Fenitrothion	30	108.6 ± 19	114.9 ± 20	110.9 ± 19
Fenobucarb	30	89.8 ± 19	88.0 ± 19	84.6 ± 18
Fenoxycarb	30	86.1 ± 15	100.0 ± 17	94.9 ± 16
Fenpropimorph	30	88.0 ± 20	89.1 ± 21	84.8 ± 20
Fensulfothion	30	112.9 ± 16	115.5 ± 16	111.1 ± 15
Fenthion	30	114.8 ± 20	104.8 ± 18	97.3 ± 17
Fenthion-sulfoxid	30	112.3 ± 19	113.2 ± 20	109.2 ± 19
Fipronil	30	117.8 ± 6	113.4 ± 6	106.0 ± 5
Flamprop-*M*-isopropyl	30	115.3 ± 22	104.3 ± 20	102.9 ± 20
Flamprop-*M*-methyl	30	103.8 ± 17	99.3 ± 16	96.3 ± 16
Flufenacet	30	93.6 ± 24	85.9 ± 22	91.6 ± 24
Flufenoxuron	30	ND	112.5 ± 11	100.5 ± 10
Flumioxazin	30	116.9 ± 12	ND	107.1 ± 11
Fluometuron isomer	30	97.0 ± 21	96.0 ± 21	87.8 ± 19
Fluopicolide	30	102.4 ± 19	99.1 ± 19	99.1 ± 19
Fluopyram	30	97.6 ± 20	99.1 ± 20	98.5 ± 20
Fluoxastrobin	30	88.8 ± 21	98.3 ± 23	93.2 ± 22
Fluquinconazole	30	93.4 ± 12	101.8 ± 13	91.8 ± 12
Fluridone	30	94.2 ± 11	94.2 ± 11	88.5 ± 11
Flutolanil	30	100.8 ± 14	97.5 ± 14	95.2 ± 14
Flutriafol	30	114.6 ± 24	111.1 ± 24	111.1 ± 24
Fluxapyroxad	30	112.1 ± 23	110.7 ± 23	107.5 ± 22
Fonofos	30	90.2 ± 18	82.3 ± 17	82.8 ± 17
Forchlorfenuron	30	94.4 ± 16	93.2 ± 16	89.6 ± 16
Fosthiazate	30	88.1 ± 20	89.7 ± 21	86.6 ± 20
Furalaxyl	30	92.9 ± 9	93.6 ± 9	90.8 ± 9
Furathiocarb	30	80.9 ± 14	87.8 ± 15	ND
Haloxyfop	30	117.3 ± 8	109.9 ± 5	114.1 ± 7
Hexaconazole	30	98.5 ± 9	104.5 ± 10	92.0 ± 9
Hexaflumuron	30	103.3 ± 10	99.0 ± 9	109.6 ± 10
Hydramethylnon	30	ND	83.5 ± 6	94.7 ± 7
Imazalil	30	86.8 ± 8	87.2 ± 8	84.2 ± 8
Indoxacarb	30	88.9 ± 16	106.6 ± 19	103.6 ± 19
Ipconazole	30	107.7 ± 7	105.9 ± 7	85.4 ± 8
Iprodione	30	86.2 ± 9	96.8 ± 8	93.4 ± 7
Iprovalicarb isomer	30	92.6 ± 9	106.4 ± 10	96.2 ± 9
Isazofos	30	88.1 ± 2	85.6 ± 2	82.7 ± 2
Isoprocarb	30	94.9 ± 5	94.6 ± 5	91.6 ± 5
Isoprothiolane	30	ND	82.9 ± 14	ND
Isoproturon	30	90.2 ± 20	91.4 ± 21	87.0 ± 20
Isopyrazam	30	83.8 ± 21	85.7 ± 22	91.4 ± 23
Kresoxim-methyl	30	85.6 ± 15	90.1 ± 16	100.6 ± 18
Lenacil	30	93.3 ± 14	92.6 ± 14	90.2 ± 14
Linuron	30	98.9 ± 10	99.8 ± 10	95.4 ± 9
Lufenuron	30	116.9 ± 10	113.2 ± 10	96.2 ± 8
Malaoxon	30	80.8 ± 8	81.0 ± 8	ND
Malathion	30	102.7 ± 33	98.9 ± 32	97.6 ± 32
Mandipropamid	30	107.6 ± 16	101.0 ± 15	101.2 ± 16
Mefenacet	30	87.8 ± 16	82.9 ± 15	ND
Mepronil	30	95.9 ± 13	99.0 ± 13	93.5 ± 13
Metaflumizone	30	ND	81.5 ± 8	ND
Metalaxyl	30	96.3 ± 11	97.5 ± 11	93.4 ± 11
Metazachlor	30	87.2 ± 21	84.5 ± 20	84.1 ± 20
Metconazole	30	103.4 ± 4	100.4 ± 3	88.7 ± 9
Methabenzthiazuron	30	88.0 ± 23	88.4 ± 24	83.9 ± 22
Methiocarb	30	87.0 ± 13	87.6 ± 13	87.7 ± 13
Methoprene	30	101.5 ± 13	104.1 ± 13	ND
Methoprotryne	30	88.3 ± 5	91.6 ± 6	86.7 ± 4
Metobromuron	30	98.0 ± 10	98.5 ± 10	94.3 ± 9
Metolachlor	30	84.0 ± 9	88.0 ± 9	86.7 ± 9
Metrafenone	30	113.3 ± 16	101.9 ± 14	91.0 ± 13
Monolinuron	30	94.0 ± 7	93.3 ± 7	89.4 ± 6
Myclobutanil	30	111.9 ± 13	118.2 ± 13	108.4 ± 12
Neburon	30	99.6 ± 19	103.0 ± 20	110.1 ± 21
Novaluron	30	94.9 ± 3	101.7 ± 5	101.1 ± 5
Nuarimol	30	117.4 ± 5	ND	113.3 ± 5
Oxycarboxin	30	ND	80.3 ± 11	ND
Paclobutrazol	30	109.1 ± 19	110.4 ± 19	107.2 ± 19
Paraoxon-methyl	30	93.0 ± 17	93.9 ± 17	86.6 ± 16
Parathion	30	108.9 ± 22	104.5 ± 21	99.9 ± 21
Penconazole	30	91.1 ± 23	86.2 ± 22	82.2 ± 21
Phenmedipham	30	95.8 ± 13	91.7 ± 13	94.8 ± 13
Phenthoate	30	82.8 ± 21	86.4 ± 22	87.9 ± 22
Phosalone	30	112.6 ± 33	94.8 ± 28	93.8 ± 28
Phoxim	30	84.0 ± 7	90.7 ± 7	84.9 ± 7
Picoxystrobin	30	88.5 ± 23	116.7 ± 30	106.2 ± 27
Piperonyl butoxide	30	87.8 ± 24	84.9 ± 24	ND
Pirimiphos-ethyl	30	89.7 ± 13	84.9 ± 12	ND
Pirimiphos-methyl	30	81.3 ± 17	83.0 ± 17	ND
Prochloraz	30	90.7 ± 12	98.6 ± 13	85.3 ± 11
Profenofos	30	101.8 ± 12	92.2 ± 11	115.1 ± 14
Promecarb	30	86.7 ± 23	87.9 ± 23	85.3 ± 23
Prometon	30	84.1 ± 15	86.0 ± 15	81.1 ± 14
Prometryne	30	81.9 ± 20	m ± 20	ND
Propachlor	30	92.1 ± 7	92.8 ± 7	89.5 ± 6
Propanil	30	108.1 ± 3	109.2 ± 3	100.6 ± 3
Propaquizafop	30	115.8 ± 18	107.1 ± 16	87.5 ± 13
Propetamphos	30	103.9 ± 4	98.7 ± 3	88.5 ± 3
Propiconazole	30	101.5 ± 7	96.5 ± 7	91.4 ± 6
Propyzamide	30	102.4 ± 13	100.5 ± 13	99.1 ± 12
Prothioconazole-desthio	30	81.0 ± 4	83.7 ± 4	81.1 ± 4
Pyraclofos	30	114.9 ± 15	108.2 ± 14	95.6 ± 12
Pyraclostrobin	30	91.8 ± 20	91.3 ± 20	83.1 ± 18
Pyraflufen-ethyl	30	110.9 ± 18	112.0 ± 18	109.7 ± 18
Pyrazophos	30	108.4 ± 8	105.9 ± 7	109.3 ± 8
Pyrethrins	30	ND	ND	103.7 ± 27
Pyridaphenthion	30	113.5 ± 16	112.3 ± 16	105.2 ± 15
Pyrimethanil	30	82.0 ± 10	85.6 ± 11	ND
Quinalphos	30	100.3 ± 17	93.9 ± 16	88.5 ± 15
Rotenone	30	104.6 ± 15	105.6 ± 15	102.2 ± 15
Secbumeton	30	83.2 ± 8	85.1 ± 8	80.6 ± 8
Siduron	30	97.3 ± 17	97.9 ± 17	95.7 ± 17
Spinetoram	30	118.9 ± 10	117.6 ± 10	102.2 ± 9
Spinosad (spinosyn A)	30	89.0 ± 2	95.1 ± 3	97.1 ± 3
Spirotetramat	30	ND	118.9 ± 3	116.3 ± 3
Spiroxamine isomer	30	87.3 ± 18	88.4 ± 18	ND
Sulfosulfuron	30	106.4 ± 20	108.0 ± 20	106.0 ± 19
Sulfotep	30	ND	84.8 ± 15	88.0 ± 16
Sulprofos	30	98.9 ± 9	88.9 ± 8	83.2 ± 8
Tebuconazole	30	93.7 ± 15	87.6 ± 14	88.0 ± 15
Tebufenozide	30	93.9 ± 6	89.8 ± 5	100.7 ± 8
Tebufenpyrad	30	100.6 ± 18	91.2 ± 16	83.0 ± 15
Tebuthiuron	30	80.4 ± 10	80.9 ± 10	ND
Teflubenzuron	30	ND	86.9 ± 20	85.1 ± 19
Temephos	30	119.7 ± 21	ND	108.9 ± 19
Terbufos sulfoxid	30	ND	88.4 ± 10	ND
Terbumeton	30	85.5 ± 10	87.3 ± 10	81.9 ± 9
Terbutryn	30	ND	82.0 ± 23	ND
Tetraconazole	30	112.9 ± 17	110.1 ± 17	104.2 ± 16
Tetramethrin	30	94.7 ± 20	98.2 ± 21	93.3 ± 20
Thidiazuron	30	119.0 ± 20	117.3 ± 20	118.8 ± 20
Thiobencarb	30	89.3 ± 9	M ± 8	ND
Thiodicarb	30	ND	82.2 ± 12	ND
Thiophanate-methyl	30	109.3 ± 2	115.7 ± 2	118.6 ± 3
Tolclofos-methyl	30	84.3 ± 3	92.6 ± 3	86.3 ± 3
Tolfenpyrad	30	105.7 ± 9	99.6 ± 7	84.1 ± 9
Triadimefon	30	117.6 ± 14	117.8 ± 14	116.6 ± 13
Triadimenol	30	111.8 ± 12	116.0 ± 14	109.0 ± 14
Triazophos	30	100.9 ± 12	93.1 ± 11	88.7 ± 11
Trifloxystrobin	30	110.1 ± 33	99.1 ± 30	91.1 ± 27
Triflumizole	30	85.7 ± 16	109.0 ± 20	ND
Triflumuron	30	83.9 ± 19	92.2 ± 21	90.1 ± 20
Trinexapac-ethyl	30	117.0 ± 13	113.6 ± 13	109.8 ± 12
Triticonazole	30	ND	118.4 ± 18	ND
Zoxamide	30	85.4 ± 12	93.2 ± 13	91.9 ± 13

It is crucial to mention that HAAs should not be renewed or reused since they react with the food sample's fat matrix and will precipitate and be discarded at the conclusion of the analysis. Rewashing or eliminating the fat content with an organic solvent may cause structural damage to HAAs or eliminate it along with fat molecules.

## 4. Conclusion

In this project, alginic acid was chemically modified using an eco-friendly esterification procedure with three alcohols (methanol, butanol, and octadecanol) to obtain hydrophobic alginic acids (HAAs). The synthesis of HAAs occurred and was confirmed by FT-IR, TGA, XRD, and SEM. Subsequently, the performance of these biopolymers was evaluated by measuring their ability to remove the fat content of a fatty food sample. The fat removal percent was calculated for each HAA. The highest percent was recorded for HAA-C18, but all materials were successful, with approximate values between 77% and 83%. The fabricated HAAs were used in a real fatty food sample analysis to determine multi-pesticide residues. All HAAs were satisfactorily able to remove fat from a final extractor, and pesticide compounds were successfully recovered. HAA-C4 provided fewer missing multi-class pesticide compounds compared with HAA-C1 and HAA-C18 due to its medium hydrophobicity. Therefore, HAAs are developable and a promising and efficient method to be used in food analysis applications.

## Disclaimer

“The views expressed in this paper are those of the author(s) and do not necessarily reflect those of the SFDA or its stakeholders. Guaranteeing the accuracy and the validity of the data is a solely the responsibility of the research team”.

## Conflicts of interest

There are no conflicts to declare.

## Supplementary Material
